# Dr. Buck on the Use and Abuse of Medicine

**Published:** 1845-06

**Authors:** 


### PRACTICAL MEDICINE, &c.

*Dr. Buck on the Use and Abuse of Medicine.*—M. Trousseau (now professor of Materia Medica in the Medical School of Paris,) in a work entitled "A Treatise on Therapeutics and Materia Medica," says, in vol. II, page 217: "We saw, at the Hospital of Tours, a young nun *(une jeune religieuse)* remain insane *(folle)* during one day, in consequence of having taken, at one time, 24 grains of sulph. quinine. One day, by our advice, a tailor of the 2d Reg't. of Carbineers, took, at at one time, 48 grains of sulph. of quinine, for the relief of asthma, which returned every day at a fixed hour. Four hours after taking the medicine, he experienced noise in the ears, dulness of the senses *(étourdissement)*, vertigo and horrible vomiting. We saw him seven hours after the administration of the quinine; he was blind and deaf, his mind wandered, and he could not walk, so great was the vertigo he experienced; at each moment he vomited floods (*flots*) of bile; in a word, he was under the influence of quinine intoxication. These accidents, for which we did not prescribe any active medicine, yielded spontaneously in the course of the night. When, instead of giving a dose as large as that which had been taken by this patient, we gave a smaller one of 15, 20 and 25 grains in the day, we do not avoid all the accidents:

a dulness of hearing was the one especially of which the greater part of the patients complained; it seems to them that they hear at a distance," &c.

At page 218, he says: "Daily observation, says Brittoneau, proves that bark, given in large doses, produces, in many persons, a very well remarked febrile movement." * * * * "Most frequently tinnitus aurium, deafness and a kind of drunkenness precede the invasion of this fever." "Sulphate of quinine often produces diarrhœa. Applied locally, by the endermic method, it irritates, produces considerable local pain, and there are manifested undoubted signs of inflammation,"* &c. &c.

At the opening of the Faculty of Medicine of Paris on the 15th Dec., 1842, a letter was received from Dr. Rognetta, an Italian oculist in Paris, claiming the priority in the use of quinine in rheumatism, for Rasori. He says: "In a series of experiments lately introduced by Dr. Giacomini, he found that the solution of the sulphate of quinine, administered in large doses, determined a general hyposthenic intoxication, which was only dissipated by the use of mercury, opium, canella," &c. Dr. Rognetta thinks, with the Italian physicians, "that the limits of tolerance should not be exceeded, and that beyond this, a species of poisoning may be induced, known by deafness, blindness, hallucinations, hæmaturia," &c.

The same letter, addressed to the Editor of the Examiner, says, "five accidents have lately resulted from this practice, of which two have terminated fatally. Two of these occurred at the hospital Cochin, and one at La Charité. One patient died immediately after swallowing a single dose of seventy-six and a half grains of the salt. At the Hospital Cochin, a woman laboring under chronic rheumatism, or a disease so called, succumbed soon after the administration of a large dose of the quinine. A young girl, after the use of the same medicine, became affected with amaurosis, which has already existed for three weeks, in spite of the most appropriate and energetic treatment. The patient at La Charité experienced, at first, pain in the head, then tinnitus and general agitation, and finally violent delirium terminating in coma. From this condition she recovered only by the employment of the most active and violent remedies, and after all hope of safety had been abandoned. Except some grave complication occurs with its ordinary termination, acute rheumatism, we all know, is rarely fatal. When death occurs, it is from a phlegmasia of serous or fibro-serous tissue, and more particularly those of the heart. How, then, does it happen, that just at the moment these huge doses of sulphate of quinine become fashiona-

* For this French authority and some others, I am indebted to my friend Dr. Johnston, whose scientific attainments, professional ardor and punctuality, have been highly serviceable to the Medical Department of the National Institute.

ble, acute rheumatism should become so fatal a disease? Is it a singular and inflexible coincidence? What are the symptoms which precede death in these cases? The usual ones which follow the exhibition of over-doses of this medicine, and none other. Is not a demand for future observations a demand for fresh victims? Are there not simpler and surer means of discovering the cause of this extraordinary mortality? Should not humanity and reason dictate that we should suspend this new treatment, and then see if mortality persists," &c.

In a discussion which followed the reading of a paper on quinine, M. Piorry stated, "that in typhus fever, with engorgement of the spleen, he had seen quinine prove serviceable, which had not been the case when the fever was unaccompanied by splenic lesion." "M. Martin Solon, who had employed the remedy under the personal inspection of Sig. Broqua at the Hospital Beaujon, admitted that in cases in which the fever assumed a remittent type, quinine was useful, but the remittent typhus was rare—at least in Paris. Of five severe cases of typhus fever in which quinine had been given, death had resulted in three instances; and in the two others recovery had only taken place after a considerable lapse of time, and without any evidence to show that the sulphate of quinine had been the means of hastening it" (the recovery.) "In the *post-mortem* examinations of the subjeets who died (says M. Martin Solon,) I failed to detect any particular alteration that I could fairly attribute to the large doses of the sulphate; it had passed in a manner imperceptibly through the stomach and intestines. A symptom which I discovered in those who recovered from the disease, was a remarkable depression of the circulation. In short, I consider the advantages attributed to the sulphate (quinine) more than doubtful. Much doubt was afterwards expressed by several members of the Academy as to the innocuity of large doses of quinine or its sulphate; but finally, the terms of the report were adopted, and the memoir was shelved by a majority of voices." (See London Lancet, Feb. 25th, 1843.)

In the French Academy of Medicine, "M. Guenneau de Mussy read a report on the different papers which had been forwarded to the Academy, on the treatment of acute rheumatism by sulphate of quinine in high doses. After a careful consideration of all the points connected with this disputed question, the committee conclude that the sulphate of quinine should not be prescribed in the high doses of four or six scruples, recommended by M. Briquet; and, 2ndly, that the same therapeutic effects may be obtained by the ordinary doses of the remedy."

In the Provincial Medical Journal, Dec. 23, 1843, a young lady, aged 18, of a delicate constitution and nervous temperament, was under the treatment of a medical friend of mine for severe hysterical symptoms. Almost every evening she had repeated fits—epileptic in appearance, although decidedly hysterical in character. The remedy administered was quinine with sulphuric acid; she began with six grains per diem, which was soon increased to ten, and afterwards to twenty. When she had been taking the medicine for about a fortnight, she received a six-ounce bottle containing eight drachms of quinine in solution, with an ounce and a half of dilute sulphuric acid, of which she was directed to take one teaspoonful at a dose, in a wineglassful of water. Not regarding the directions, however, she poured out a wineglassful—about one third of the bottle—and swallowed it. Her first impression was one of extreme acidity of the mouth and a most disagreeable sensation about the teeth. This was followed by nausea and extreme giddiness, and tendency to stupor. Her friends, believing that she had taken a narcotic poison, insisted on making her walk up and down, after the manner generally recommended for narcotic poisoning. After this had been kept up for some time, the stupor abated and she passed into a state of semi-consciousness, with the feeling as if she was obliged to keep moving. She was intensely thirsty, and drank a great deal of water. But all the bad symptoms gradually subsided without medical aid, and did not return for a very long time afterwards."

In the "American Journal of Medical Sciences," for January, 1844, it is stated that "dogs, poisoned with sulphate of quinine, showed distinct fluidity of the blood and morbid engorgement of the parenchyma of the lungs. M. Metier cautions against administering the large doses of quinine that have been in use."

In the April No. of the same Journal, page 498, we have the following case: "M. Recamier ordered for a man 26 years of age, admitted into the Hotel Dieu, 27th Nov., 1842, laboring under acute rheumatism, 48 grains of sulph. of quinine in 12 powders, to be taken every hour. The next day 72 grains were ordered, six to be taken every hour; but after the eighth dose, the patient was suddenly seized with a violent agitation, followed by furious delirium, and died in a few hours. On examination, evidences of severe inflammation of the cerebral membrane were discovered."

Since I commenced the investigation of this subject, I have found so many authorities opposed to the administration of large doses of quinine, that I should fill a volume, if I detailed them all at length. I have given fair specimens of them, without suppressing anything, in the cases cited, that would seem to favor it.—*Boston Medical and Surgical Journal.*

